# Phosphoproteomic analysis reveals *Smarcb1* dependent EGFR signaling in Malignant Rhabdoid tumor cells

**DOI:** 10.1186/s12943-015-0439-5

**Published:** 2015-09-15

**Authors:** Jonatan Darr, Agnes Klochendler, Sara Isaac, Tami Geiger, Amir Eden

**Affiliations:** Department of Cell and Developmental Biology, The Alexander Silberman Institute of Life Sciences, The Hebrew University of Jerusalem, Jerusalem, Israel; Department of Human Molecular Genetics and Biochemistry, Sackler Faculty of Medicine, Tel Aviv University, Tel Aviv, Israel

**Keywords:** Rhabdoid, MRT, AT/RT, Phosphoproteomics, EGFR, Gefitinib, Lapatinib

## Abstract

**Background:**

The SWI/SNF ATP dependent chromatin remodeling complex is a multi-subunit complex, conserved in eukaryotic evolution that facilitates nucleosomal re-positioning relative to the DNA sequence. In recent years the SWI/SNF complex has emerged to play a role in cancer development as various sub-units of the complex are found to be mutated in a variety of tumors. One core-subunit of the complex, which has been well established as a tumor suppressor gene is *SMARCB1* (*SNF5*/*INI1*/BAF47). Mutation and inactivation of *SMARCB1* have been identified as the underlying mechanism leading to Malignant Rhabdoid Tumors (MRT) and Atypical Teratoid/Rhabdoid Tumors (AT/RT), two highly aggressive forms of pediatric neoplasms.

**Methods:**

We present a phosphoproteomic study of *Smarcb1* dependent changes in signaling networks. The SILAC (Stable Isotopic Labeling of Amino Acids in Cell Culture) protocol was used to quantify in an unbiased manner any changes in the phosphoproteomic profile of *Smarcb1* deficient murine rhabdoid tumor cell lines following *Smarcb1* stable re-expression and under different serum conditions.

**Results:**

This study illustrates broad changes in the regulation of multiple biological networks including cell cycle progression, chromatin remodeling, cytoskeletal regulation and focal adhesion. Specifically, we identify *Smarcb1* dependent changes in phosphorylation and expression of the EGF receptor, demonstrate downstream signaling and show that inhibition of EGFR signaling specifically hinders the proliferation of *Smarcb1* deficient cells.

**Conclusions:**

These results support recent findings regarding the effectivity of EGFR inhibitors in hindering the proliferation of human MRT cells and demonstrate that activation of EGFR signaling in Rhabdoid tumors is *SMARCB1* dependent.

**Electronic supplementary material:**

The online version of this article (doi:10.1186/s12943-015-0439-5) contains supplementary material, which is available to authorized users.

## Introduction

The SWI/SNF ATP dependent chromatin remodeling complex is a multi-subunit complex, conserved in eukaryotic evolution, that facilitates nucleosome re-positioning relative to the DNA sequence [1]. The SWI/SNF complex has been found to play a role in fundamental cellular functions such as transcriptional regulation, DNA replication and DNA repair, but is mainly regarded to as a broad transcriptional co-activator / co-repressor [2].

In recent years various deep sequencing studies have demonstrated repeating mutations in sub-units of the SWI/SNF complex across various types of tumors [3, 4]. One core-subunit of the complex, which has been well established as a tumor suppressor gene is *SMARCB1* (*SNF5*/*INI1*/BAF47). As more and more tumors are deep sequenced, mutations in *SMARCB1* are found across a growing spectrum of cancers. More specifically, inactivating mutations of *SMARCB1* are found in all Malignant Rhabdoid Tumors (MRT) and Atypical Teratoid/Rhabdoid Tumors (AT/RT), two highly aggressive forms of pediatric neoplasms [5]. In spite of significant progress in treatment over recent years, long-term prospects for MRT and AT/RT patients remain poor as the tumors demonstrate relative resistance to conventional chemotherapy and radiotherapy and tumor resection is in many cases not possible [6, 7].

MRT which manifests in the kidney and AT/RT of the central nervous system are unique in that apart from the *SMARCB1* locus they show unusually low mutation rate. Several recent deep sequencing studies have revealed the poor mutational landscape of these tumors [8–11]. This finding suggests that *SMARCB1* inactivation alters multiple pathways that promote cellular transformation, and results in the simultaneous acquisition of the various hallmarks of a transformed cancer cell [12] through a singular mutation.

We have been studying *SMARCB1* associated transformation using cell lines derived from rhabdoid tumors which developed in *Smarcb1* heterozygous *p53* null mice [13]. These tumor cell lines show loss of heterozygosity and lack *Smarcb1*. Restoration of *Smarcb1* expression had a minor effect on cell proliferation in culture but completely ablated the tumorigenic capacity of xenografted tumor cells [14]. This result indicates that by comparing the *Smarcb1* deficient and proficient tumor cells one can define *Smarcb1* dependent changes which are functionally relevant to transformation. Using this system we previously showed that *Smarcb1* deficiency results in persistent AKT activation. Accordingly we found that *Smarcb1* deficient tumor cells are specifically vulnerable to AKT or PI3-kinase inhibition [14].

In this study we use a high throughput phosphoproteomic analysis comparing *Smarcb1* deficient and proficient tumor cells to further identify aberrant signaling associated with *Smarcb1* deficiency. We describe *Smarcb1* dependent constitutive phosphorylation of the EGFR, which is also transcriptional elevated in *Smarcb1* deficient cells and demonstrate that inhibition of the EGFR/ERBB signaling pathway inhibits proliferation of *Smarcb1* deficient tumor cells. We further identify multiple biological networks and kinases whose regulation is altered in *Smarcb1* deficient tumor cells in a *Smarcb1* dependent manner.

## Results

### Profound changes in the phosphoproteomic landscape between *Smarcb1* proficient and deficient cells

We previously reported persistent activation of AKT in *Smarcb1* deficient cells [14], yet we could not identify the cause of this *Smarcb1* dependant activation. To better characterize altered signaling pathways in *Smarcb1* deficient tumor cells, which may contribute to the transformation process and to AKT activation, we conducted an unbiased quantitative phospho-proteomic analysis designed to identify differentially phosphorylated peptides between *Smarcb1* proficient and deficient tumor cells.

**Fig. 1:**
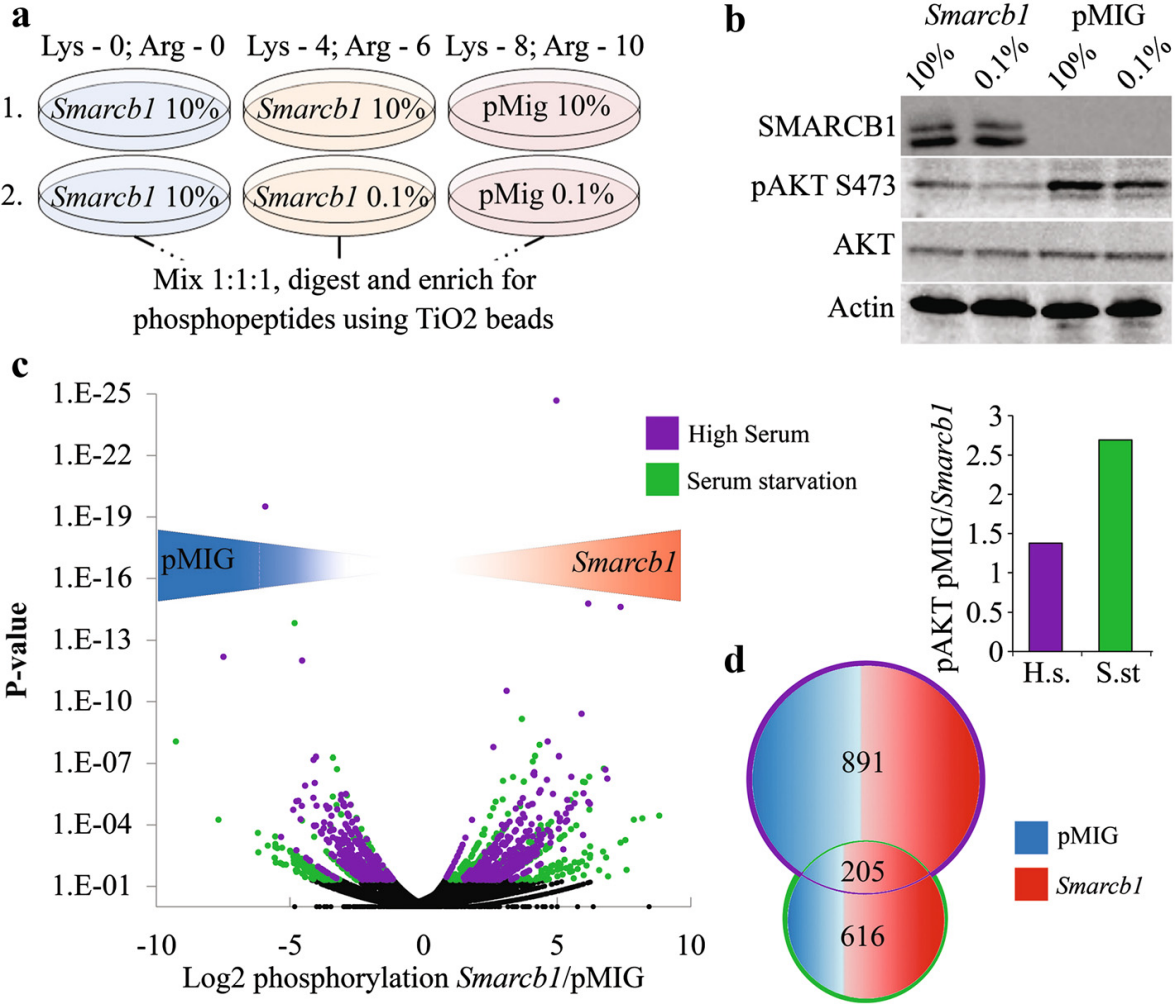
Phosphoproteomic profiling reveals robust *Smarcb1* dependent changes in protein phosphorylation. Matching *Smarcb1* proficient and deficient tumor cells were generated by re-introducing SMARCB1 (pMIG- *Smarcb1*) or an empty vector as control (pMIG) [14]. **a** The outline of the phosphoproteomic study. Two triple-SILAC experiments were conducted. The first set allowed evaluation of *Smarcb1* dependent changes when cultured in normal serum whilst the second set allowed for the evaluation under serum starvation. *Smarcb1* proficient cells grown in normal serum and light isotopic labeling were included in both sets to allow comparison between the two sets. **b** Western blot demonstrating differential AKT phosphorylation in *Smarcb1* deficient versus proficient cells. Bar graph shows quantification of western blot presented as fold change in AKT phosphorylation in pMIG/*Smarcb1* cells normalized to beta-actin. **c** Volcano plot depicting *Smarcb1* dependent changes in site phosphorylation across the two sets. X-axis is the log_2_ ratio of the abundances of specific residues between *Smarcb1* proficient and deficient cells. Negative values for highly phosphorylated in *Smarcb1* deficient cells. Y-axis is the logarithmic scale for the *P*-value of the fold change. For P.V < 0.05; Violet dataset represents residues altered in high serum; Green dataset represents residues altered under serum starvation. **d** Venn-diagram portraying the residues found to be differentially phosphorylated in a *Smarcb1*-dependent manner. Under 10 % serum, 458 residues are highly phosphorylated in *Smarcb1* proficient cells (*In red*) whilst 434 residues are highly phosphorylated in *Smarcb1* deficient cells (*In blue*). Under low serum, 384 residues (roughly two thirds) are highly phosphorylated in *Smarcb1* proficient cells, whilst 233 residues are highly phosphorylated in *Smarcb1* deficient cells. Overall 205 residues are differentially phosphorylated between *Smarcb1* proficient and deficient cells regardless of the serum conditions

The triple - SILAC (Stable Isotopic Labeling of Amino Acids in Cell Culture) protocol [15–18] was used to compare *Smarcb1* deficient and proficient tumor cells (Cell line 365 [14] containing an empty retroviral vector as control (pMIG) or pMIG-Smarcb1 respectively) (Fig. 1a, b). Because serum is a rich source for signals, we expected *Smarcb1* dependent differential activation of signaling pathways to reflect better under serum starvation conditions, as demonstrated by the differential phosphorylation of AKT (Fig. 1b, c and [14]).

All in all 10701 phosphorylation sites from 3655 distinct proteins were identified using high resolution mass spectrometric analysis. 891 sites from 510 distinct proteins were differentially phosphorylated in a statistically significant manner between *Smarcb1* deficient and proficient cells under high serum, whilst under serum starvation 616 sites from 407 distinct proteins demonstrated differential phosphorylation (*P*-value < 0.05). Overall 205 residues from 134 distinct proteins exhibited a differential phosphorylation between *Smarcb1* deficient and proficient cells regardless of the growth serum condition (Fig. 1c, Additional file 1: Table S1 and Table S2).

### Altered regulation of cell adhesion and cytoskeletal organization in *Smarcb1* deficient cells

**Fig. 2:**
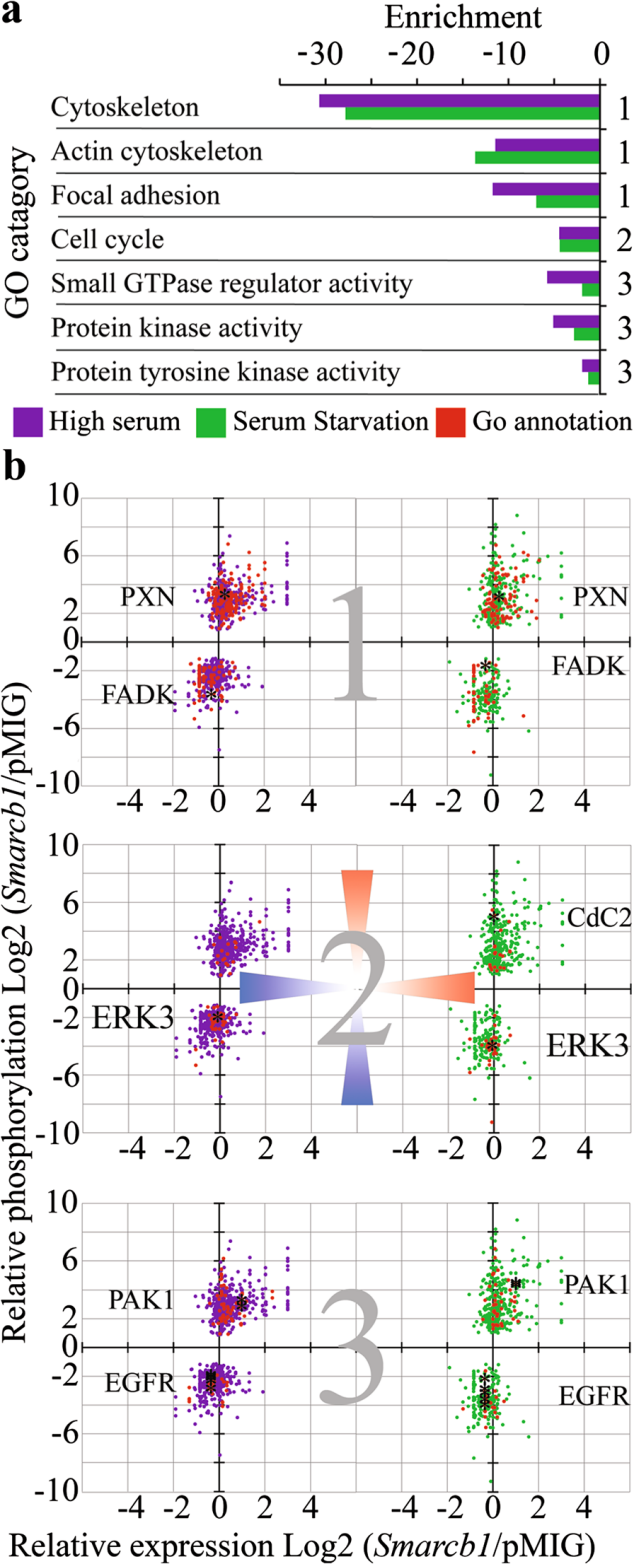
GO annotation enrichment analysis of differentially phosphorylated proteins. **a** Gene ontology (GO) annotations enrichment among proteins found to be differentially phosphorylated between SMARCB1 proficient and deficient cells. Evaluated using the David Bioinformatics tool, x-axis indicates log base for Benjamini corrected *P*-value. Violet dataset represents differentially phosphorylated peptides in high serum; green dataset represents differentially phosphorylated peptides in serum starvation. **b** Scatter plots depicting *Smarcb1* dependent differentially phosphorylated peptides and their expression level in high or low serum. Negative values denote highly expressed/phosphorylated in *Smarcb1* deficient cells, positive values denote highly expressed/phosphorylated in *Smarcb1* proficient cells. Peptides corresponding to enriched GO categories (from panel 2a) are highlighted in red. Group 1 includes GO annotations: Cytoskeleton, Actin cytoskeleton and focal adhesion. Group 2: Cell cycle. Group 3: Small GTPase regulator activity, protein kinase activity and protein tyrosine kinase activity

Across all the mentioned sets a statistically significant enrichment was found for proteins related to several GO annotations including actin cytoskeleton and focal adhesion (Fig. 2a). We previously profiled transcriptional changes brought about following re-introduction of *Smarcb1* in the same tumor cell line and found enrichment for cytoskeleton and focal adhesion categories already at transcription level [14]. However, correlating changes in phosphorylation levels with changes in levels of expression (Fig. 2b) demonstrates that only a small fraction of the changes in phosphorylation are correlated to changes in gene expression, suggesting that altered transcription accompanies altered regulation of these cellular functions.

Proteins that demonstrate differential phosphorylation between *Smarcb1* proficient and deficient cells regardless of serum conditions include Paxillin (PAX) and its binding protein Vinculin (VCL), two proteins localized to focal adhesion sites. These genes were found to be transcriptionally up-regulated in *Smarcb1* proficient cells [14]. PAX is found to be highly phosphorylated in *Smarcb1* proficient cells at residue Y118, whose phosphorylation is associated with altered cell adhesion, motility and cytoskeletal organization [19]. Moreover, Focal adhesion kinase 1 (FAK1) demonstrates elevated levels of phosphorylation in *Smarcb1* deficient cells at serine 948, whilst FAK2 has elevated levels of phosphorylation in *Smarcb1* proficient cells at serine 375. Despite the fact that the precise nature of the phosphorylation at the observed residues is unclear, these findings suggest that loss of *Smarcb1* leads to alteration in the composition and arrangement of focal adhesion sites and in the organization of the cytoskeleton. These alterations can be accompanied by deregulation of focal adhesion related signaling [20].

**Fig. 3:**
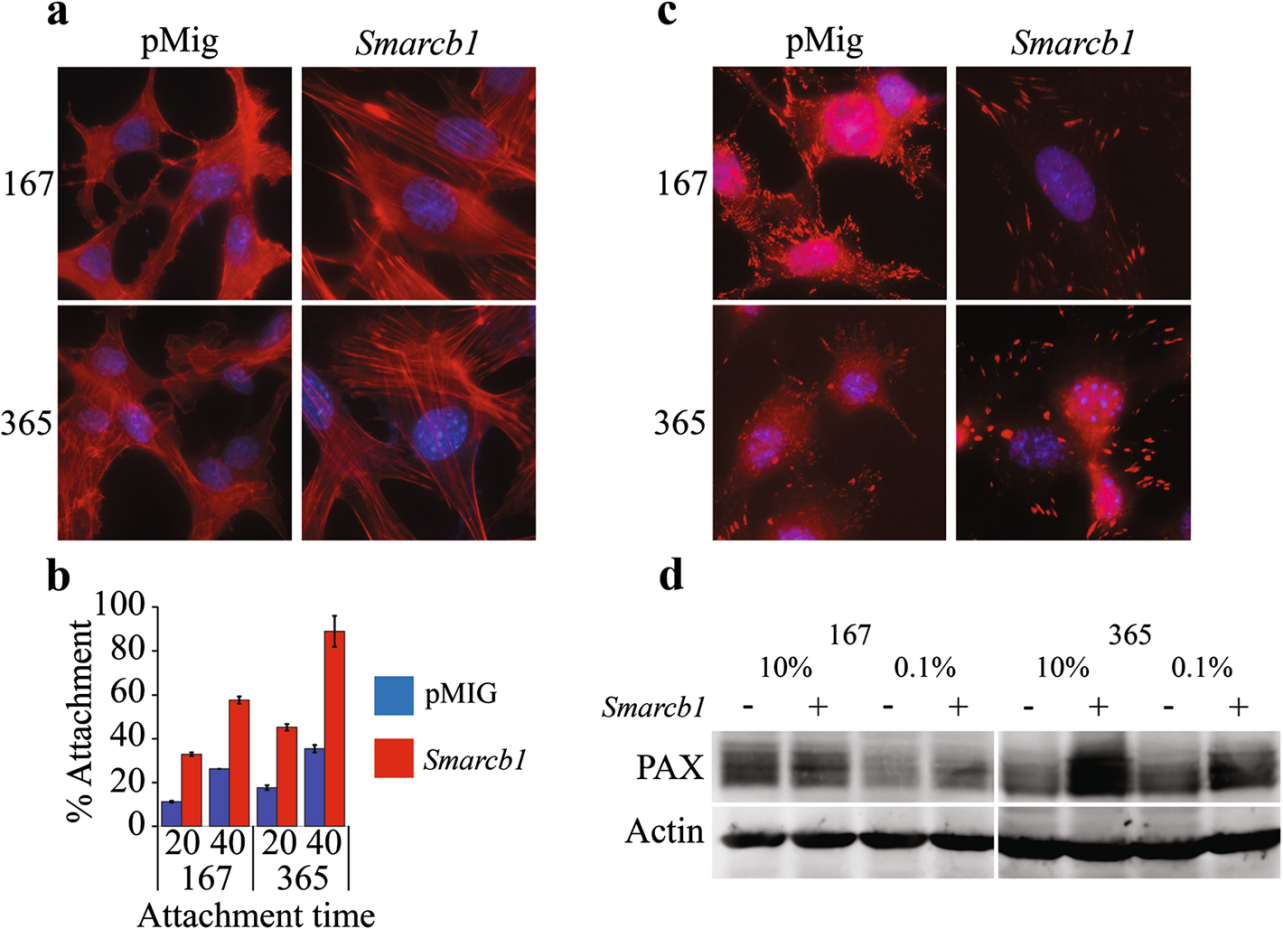
Differential morphology of *Smarcb1* proficient and deficient cell lines. **a** Whilst in *Smarcb1* proficient cell lines actin forms stress fibers across the cell, in *Smarcb1* deficient cell lines the actin is found diffused throughout the cytoplasm. In red – Phalloidin, In blue – Dapi **b** Reduced adhesiveness of *Smarcb1* deficient cell lines as evident in an adhesion assay. Cells were allowed to adhere for 20/40 min before quantification of adherent fraction was carried out as described in the materials and methods section. *T*-test; 167 20', P.V = 0.0015; 167 40', P.V = 0.0027; 365 20', P.V = 0.0008; 365 40', P.V = 0.0011. **c** Differential morphology, size and number of focal adhesion sites as visualized via Paxillin staining. In red – Paxillin, In blue – DAPI. **d** Western blot showing a slight increase in PAX following *Smarcb1* expression, consistent with available expression data

Indeed, actin staining reveals profound changes in cytoskeletal organization between *Smarcb1* proficient and deficient cells. Whilst *Smarcb1* proficient cells exhibit actin stress fibers, *Smarcb1* deficient cells lack stress fibers and the actin seems diffused throughout the cytoplasm (Fig. 3a). This *Smarcb1* dependent remodeling of the actin cytoskeleton was evident in an additional *Smarcb1* deficient murine MRT derived cell line; 167 (Fig. 3a). Adhesion assay, which assesses adhesion following cell re-plating [21], showed that *Smarcb1* deficient cells adhere less efficiently than their *Smarcb1* proficient counterparts (Fig. 3b), indicating a defect in focal adhesion. In accordance with this last finding, Paxillin immunostaining reveals gross changes in adhesion site size, number and appearance between *Smarcb1* deficient and proficient cells (Fig. 3c). Paxillin itself is moderately accumulated in *Smarcb1 *proficient cells (Fig. 3d), as expected in light of the transcriptional up-regulation. All together, these results show that loss of *Smarcb1* results in transcriptional and post-transcriptional deregulation of processes related to the actin cytoskeleton and to focal adhesion.

### Altered activation of several kinases is reflected in the phosphoproteomic data

Differential phosphorylation profiles may reflect changes in the activity of kinases and/or phosphatases between *Smarcb1* deficient and proficient cells. We therefore attempted to determine whether targets of specific kinases are over-represented in one condition over another. Using the kinase target database from Phosphositeplus [22] we constructed sets of known and characterized kinase targets at residue level for each kinase and applied the GSEA algorithm to test for enrichment of any such set in our phosphoproteomic data. Although the information linking kinases to their target sites, particularly in mouse, is very limited, this analysis identified the expected enrichment for Cyclin dependent kinase (CDK1) targets and for AKT1 targets among residues that were phosphorylated in high serum compared to serum starvation conditions in *Smarcb1* proficient cells (False Discovery Rate (FDR) = 0.05 for CDK1; FDR = 0.06 for AKT1). Application of the same approach to the *Smarcb1* dependent phosphorylation under serum starvation revealed that peptides phosphorylated in *Smarcb1* deficient tumor cells are enriched for targets of AKT, a result consistent with our previous findings demonstrating activation of AKT in the same *Smarcb1* deficient cell line used for the phosphoproteomic analysis. This activation was found to persist in serum starvation and result in phosphorylation of AKT targets such as ribosomal protein S6 [14]. Differentially phosphorylated Erk1/2 targets did not pass the statistical significance threshold as individual peptides, but as a group, were significantly overrepresented among peptides that showed elevated phosphorylation in *Smarcb1* deficient cells (Table 1). These results are consistent with elevated phosphorylation of ERK1/2 proteins at key residues in the *Smarcb1* deficient cells. (Y205 in ERK1 and T183 in ERK2 [23, 24]) in *Smarcb1* deficient cells under serum starvation found by manual examination of the data.

**Table 1:** Kinases for which phosphorylated target sites were found enriched in *Smarcb1* deficient cells. The table depicts kinases, their target residues and relative abundances of the phosphorylated peptide in SMARCB1 proficient/deficient cells (given as the log_2_ ratio). Kinase targets were defined as described in Materials and methods section. Enrichment for targets was assessed using the GSEA algorithm [53, 54]. Calculated Normalized Enrichment score for kinase targets (KS test): ERK2 = 1.85; ERK1 = 1.66; AKT1 = 1.36; JNK1 = 1.41. Calculated false discovery rate: ERK2 = 0.01; ERK1 = 0.032; AKT1 = 0.223; JNK1 = 0.205

Kinase	Target	Residue	Log2 (SMARCB1/pMIG)
	Cdc25b	S351	−1.12
	Tsc2	S939	−1.65
AKT1	Gsk3b	S9	−1.69
	Bad	S155	−2.21
	Rps6	S236	−2.28
	Acly	S455	−2.31
	Rell1	T262	−1.20
	Dcp1a	S335	−1.23
	Dcp1a	S339	−1.23
	Junb	S256	−1.35
	Mybbp1a	S1280	−1.36
	Ugdh	T474	−1.38
	Supv3l1	S725	−1.52
ERK2	Rps6ka1	S369	−1.78
	Dennd4c	S1270	−2.04
	Ndel1	T219	−2.06
	Atf2	T53	−2.08
	Tpx2	T369	−2.39
	Jun	S246	−2.87
	Ahnak	S2985	−3.08
	Ahnak	S4879	−3.39
	Egfr	T695	−3.99
	Pxn	S83	−1.18
	Junb	S256	−1.35
	Rps6ka1	S369	−1.78
ERK1	Ndel1	T219	−2.06
	Ranbp3	S58	−2.07
	Atf2	T53	−2.08
	Jun	S73	−2.76
	Jun	S63	−3.69
	Atf2	T51	−2.08
JNK1	Atf2	T53	−2.08
	Jun	S73	−2.76
	Jun	S63	−3.69

Further analysis of the phosphoproteomic data revealed additional kinases whose statistically significant differential phosphorylation levels would suggest altered activation state. These include activation of PKACA through phosphorylation of T198 [25] in *Smarcb1* proficient cell lines and phosphorylation in JNK1 Y185 [23, 24] and its targets (Table 1) in *Smarcb1* deficient cell lines (Additional file 1: Table S1). Moreover, we find persistent phosphorylation of EGFR Y1197 in *Smarcb1* deficient cells. Tyrosine 1197 is an autophosphorylation site of the EGFR associated with enzymatic activation.

### Differential response to serum reveals altered regulation of ErbB signaling in *Smarcb1* deficient tumor cells

**Fig. 4:**
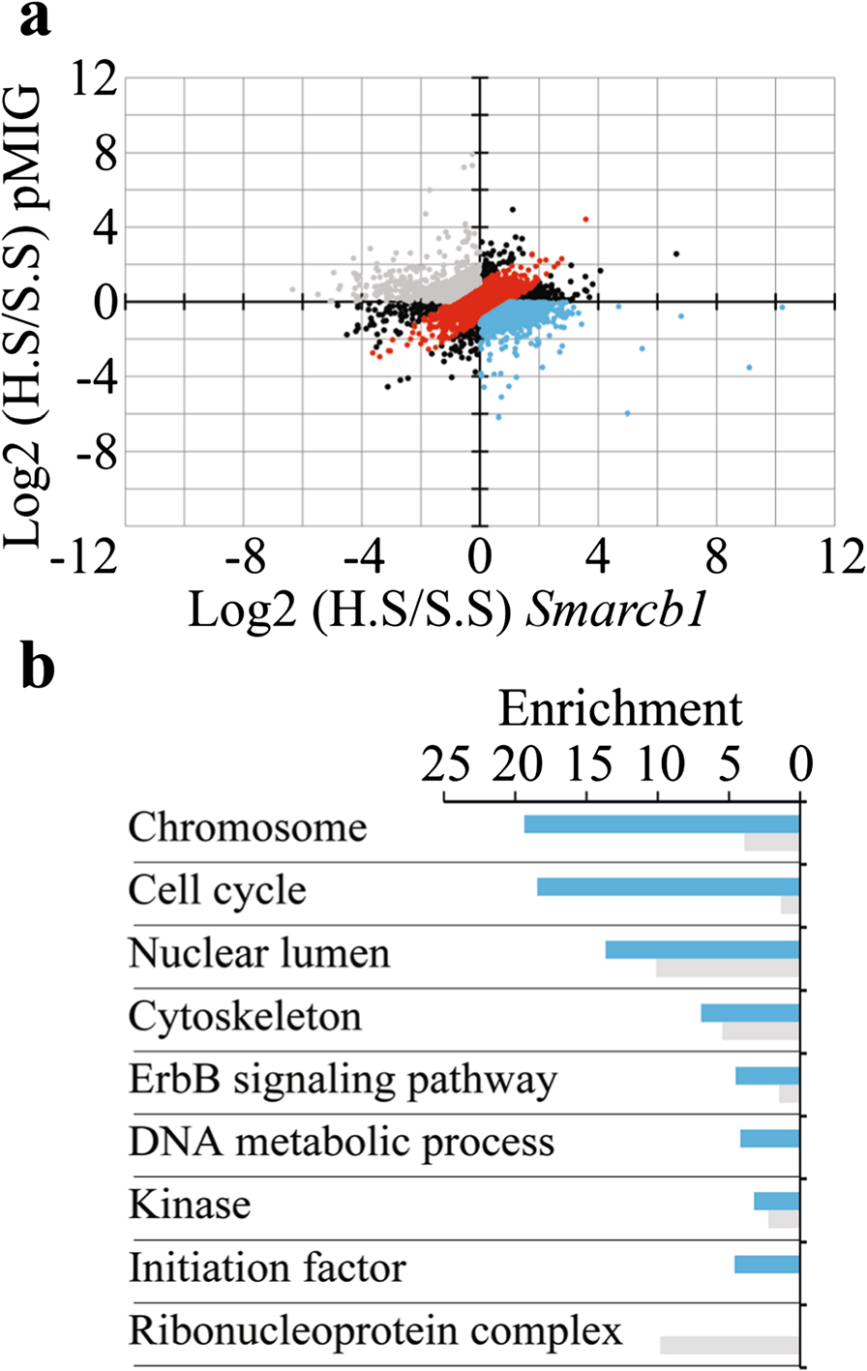
Different response to serum withdrawal between *Smarcb1* proficient and deficient cells. **a** Scatter plot depicting each peptides response to serum withdrawal (*N* = 5518 peptides). X-axis: log 2 (high serum/serum starvation) ratio of phosphopeptide abundance in *Smarcb1* proficient cells. Y-axis: log 2 (high serum / serum starvation) ratio of phosphopeptide abundance in *Smarcb1* deficient cells. Red – correlated change for *Smarcb1* proficient and deficient cells. Blue and gray – anti-correlated peptides analyzed with STRING v9.1. **b** GO annotation enrichment for networks identified using STRING v9.1. Colors correlate to panel a. X-axis represents log base for Benjamini corrected *P*-value

We next examined the differential response to serum withdrawal between *Smarcb1* proficient and deficient cells. Phosphorylation sites that were regulated in a coordinated manner in *Smarcb1* deficient and proficient cells upon serum withdrawal were excluded (Fig. 4a – red dots). Of the remaining peptides, we focused on sites whose phosphorylation level in response to serum withdrawal was the most distinct between the *Smarcb1* deficient and proficient cells (Fig. 4a – in blue and gray). The fact that few of the phosphorylation sites revealed in the analysis had any known biological effect limited our ability to deduce any functional significance from the data. Therefore, we focused on the affected proteins and used STRING v9.1 [26] to explore protein neighborhoods (high confidence physical and functional interactions) and define functional protein association networks which are differentially regulated between *Smarcb1* proficient and deficient cells upon serum withdrawal (Additional file 2: Figure S1). Several functional networks were identified for proteins, which remain phosphorylated in *Smarcb1* deficient cells upon serum withdrawal but lose their phosphorylation in *Smarcb1* proficient cells (blue group). These are enriched for proteins localized to the nuclear lumen and chromosome in addition to the cytoskeleton. More striking however is the enrichment in proteins regulating cell cycle, transcriptional initiation and the ErbB signaling pathway. In contrast, for proteins that lose phosphorylation specifically in *Smarcb1* deficient cells upon serum withdrawal (gray group), we find functional networks that are enriched for proteins localized to the nuclear lumen and function in RNA splicing and processing (Fig. 4b and Additional file 2: Figure S1). Cell cycle regulators include CDK1 which demonstrates differential phosphorylation of T14 related to cell cycle regulation. Chromatin modifying proteins include ARID1A and SMARCC1 which together with SMARCB1 assemble to form the SWI/SNF complex and regulate transcription. Proteins annotated as ErbB signaling include ERK2, JUN MYC and EGFR. Consistent with this, residue T183 in ERK2 and residue S63 in JUN which are associated with induced enzymatic activity [23, 24], and residue T58 in MYC which is required for protein degradation [27], remain phosphorylated in *Smarcb1* deficient cells upon serum withdrawal. Upstream to these affects, EGFR is found to be highly phosphorylated in *Smarcb1* deficient cells deprived of serum, in residue Y1197, an autophosphorylation site associated in humans with enzymatic activation of the receptor [28].

### Differential EGFR expression and phosphorylation promotes downstream AKT activation and cell proliferation

We previously identified persistent activation of AKT in Rhabdoid tumor cells which was *Smarcb1* dependent and central for proliferation and survival of *Smarcb1* deficient tumor cells [14]. AKT can be activated through multiple pathways and part of the motivation for performing the phospho-proteomic study was to identify the origin of AKT activation. The phosphoproteomic analysis directly indicated phosphorylation of EGFR in *Smarcb1* deficient cells, and the network analysis indicated EGFR pathway to be activated in these cells. As activation of the EGFR and ErbB signaling pathway lay upstream to all the above mentioned signaling effects observed in *Smarcb1* deficient cell lines [29], we examined EGFR activation in *Smarcb1* deficient cells.

**Fig. 5:**
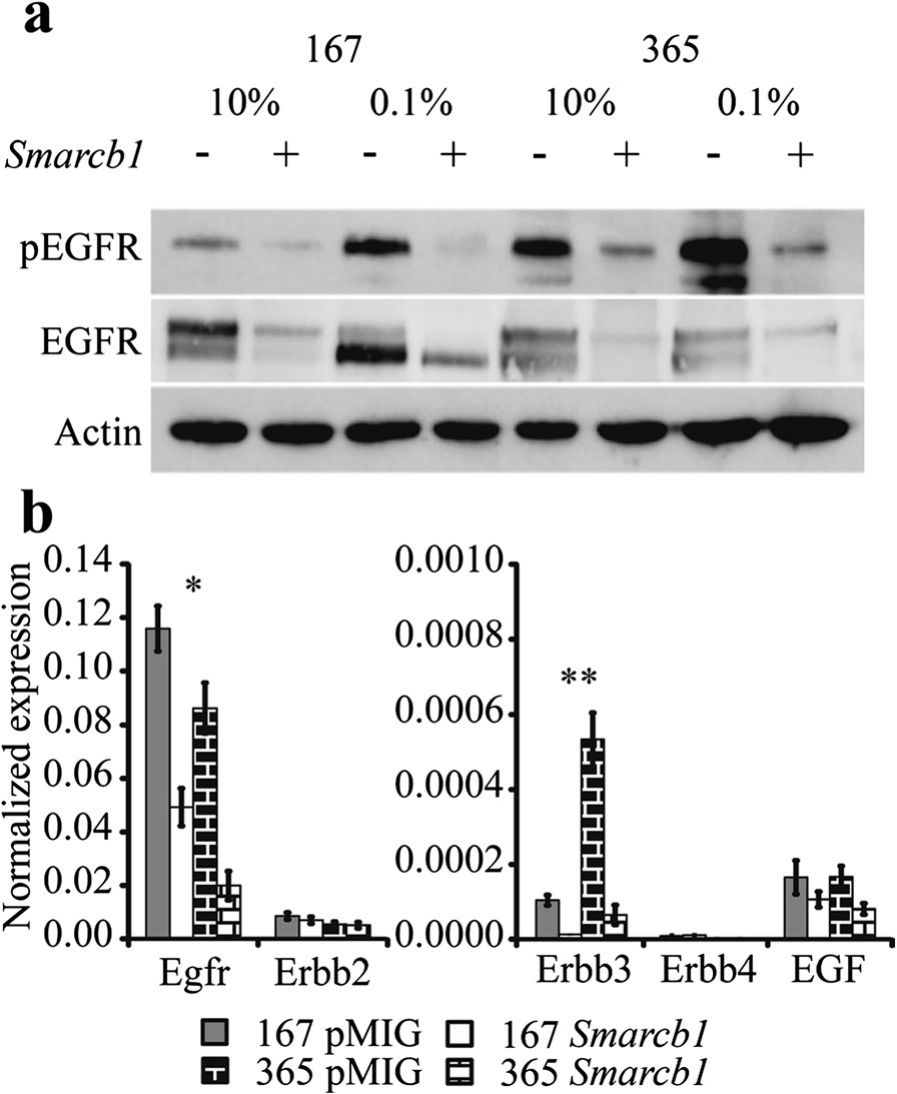
*Smarcb1* dependent EGFR phosphorylation and transcriptional de-regulation. **a** Western blot demonstrating constitutive phosphorylation of Tyr-1092 of the EGFR in *Smarcb1* deficient cells, and down-regulation of the total-EGFR protein. **b** *Smarcb1* dependent transcriptional regulation of the ErbB family receptors and the EGF ligand. Graph shows RNA levels relative to beta actin as estimated by qRT-PCR. Note that the Y axis of the right panel is two orders of magnitude lower, indicating that expression levels of ErbB3, 4 and EGF are significantly low. In both cell lines Egfr and ErbB3 showed reduced expression in *Smarcb1* proficient cells. * Fold change pMIG/*Smarcb1* 167 = 2.4, 365 = 4.5; *T*-test P.V; 167 = 0.00058, 365 = 0.0098. ** Fold change pMIG/*Smarcb1* 167 = 8.2, 365 = 6.8; *T*-test P.V; 167 = 0.0078, 365 = 0.013

Western blot analysis for an additional auto-phosphorylation site of the EGFR, residue Y1092, showed higher levels of phosphorylation in *Smarcb1* deficient tumor cells compared with *Smarcb1* proficient cells under low serum. These results reinforce the observations made in the phosphoproteomic study and indicate a *Smarcb1* dependent activation of EGFR in tumor cells (Fig. 5a).

To address the origin of EGFR activation in *Smarcb1* deficient tumor cells we considered various mechanisms that can cause aberrant activation of EGFR and downstream signaling. We find total EGFR levels to be down-regulated in *Smarcb1* proficient cells, as evident in western blot (Fig. 5a). Transcriptionally, we find Egfr to be significantly repressed in *Smarcb1* proficient relative to deficient cells (Fig. 5b). Examining the expression profile of other ErbB family members we identify ErbB3 (HER3) as an additional repressed target of SMARCB1 but the significance of this result remains to be established since ErbB3 levels are significantly lower than ErbB2 or Egfr (Fig. 5b). Though expression data from MRT and AT/RT tumors and cell lines suggests overexpression of ErbB2/Her2 relative to other central nervous system tumors [30, 31], in our system we detect no *Smarcb1* dependent change in the expression of ErbB2. ErbB4, as in most cases [32], is not expressed and unresponsive to *Smarcb1*. Egf itself is also transcriptionally unresponsive to *Smarcb1* and with very low expression level. Two additional proteins that negatively regulate EGFR (Caveolin1 [33–35] and ERRFI1 [36]) are low in *Smarcb1* deficient cells and are upregulated upon its re-introduction, but expression of either one of them in *Smarcb1* deficient cells was insufficient in diminishing EGFR or AKT activation (Additional file 2: Figure S2 and [14]).

**Fig. 6:**
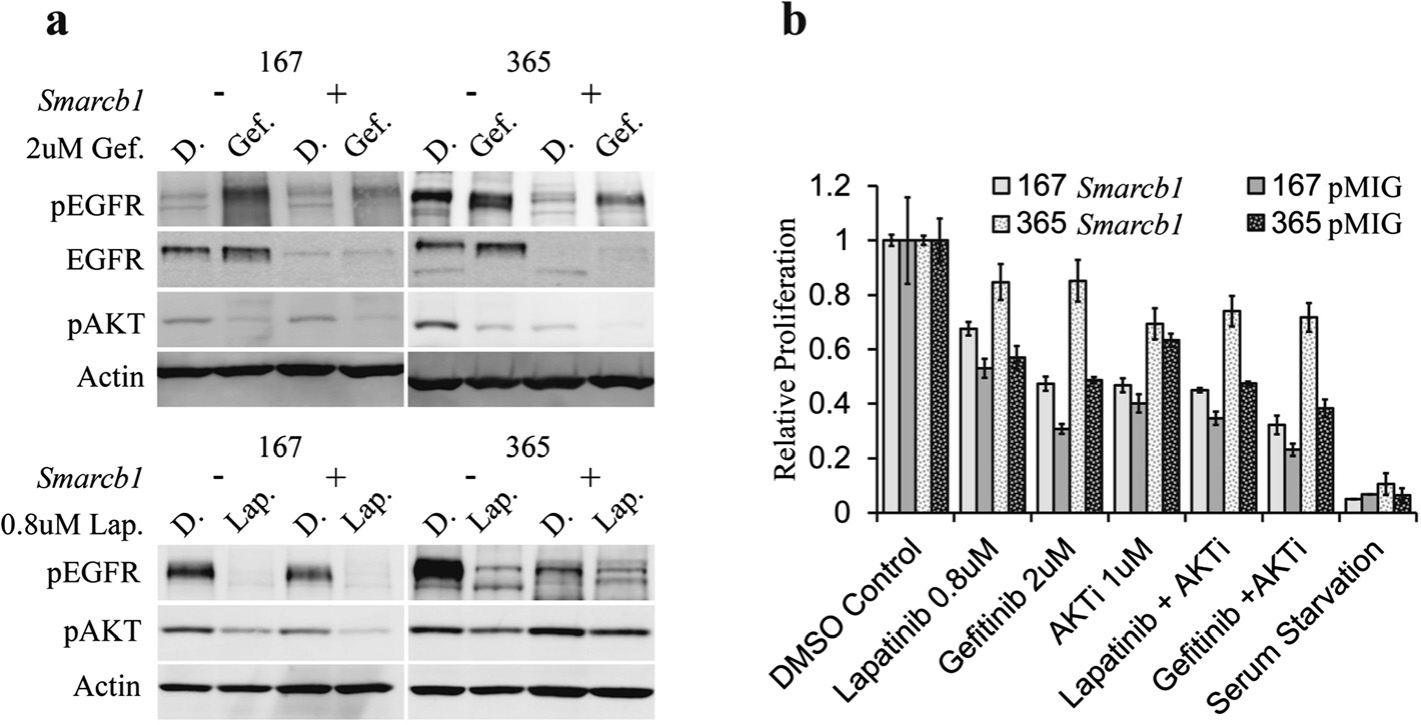
EGFR activation mediates AKT activation in *Smarcb1* deficient cells. **a** Inhibition of AKT activation upon treatment with the EGFR/HER2 inhibitor Lapatinib (Lap.) and the EGFR inhibitor Gefitinib (Gef.) versus the DMSO (D.) control. **b** WST1 proliferation assay demonstrating relative proliferation of *Smarcb1* deficient and proficient cells following a 6 day treatment with the EGFR inhibitors Lapatinib/Gefitinib, with the AKT inhibitor 1/2, dual inhibition and serum withdrawal

We next inhibited EGFR using Gefitinib, a selective inhibitor of the EGFR kinase activity, or Lapatinib a dual EGFR/ErbB2 kinase inhibitor [37]. Both treatments resulted in inhibition of AKT phosphorylation, implicating EGFR in the activation of AKT in these cells (Fig. 6a). Surprisingly, though both treatments resulted in inhibition of downstream AKT signaling, only Lapatinib treatment led to reduction in EGFR phosphorylation while Gefitinib treatment resulted in apparent elevation of EGFR phosphorylation. These results repeated in multiple experiments and may relate to differences in the preference of the molecules to altered conformations of the EGFR kinase domain [38–41], and their possible effects on the protein stability which results in the accumulation of the receptor [42]. Although the basis for this paradoxical response of EGFR to Gefitinib is unclear, both inhibitors caused reduction of AKT phosphorylation, indicating that the ErbB pathway is responsible for the persistent activation of AKT in *Smarcb1* deficient tumor cells.

Inhibition of EGFR signaling with Gefitinib or Lapatinib reduced proliferation of *Smarcb1* deficient tumor cells as demonstrated by a WST1 proliferation assay. Importantly, *Smarcb1* deficient cells demonstrated greater sensitivity to EGFR inhibitors, as their proliferation was hindered to a greater extent than their *Smarcb1* proficient counterparts (Fig. 6b). These results implicate EGFR signaling in *Smarcb1* mediated tumorigenesis and suggest that *Smarcb1* deficient cells are specifically sensitive to EGFR/ErbB2 inhibition.

## Discussion

We previously showed that re-introduction of *Smarcb1* diminishes the oncogenic capacity of *Smarcb1* deficient mouse rhabdoid tumors. By comparing *Smarcb1* deficient tumor cells with their *Smarcb1* proficient counterparts we identified persistent activation of AKT in *Smarcb1* deficient cells, which plays a key role in the survival and proliferation of these tumor cells [14]. To elucidate the source of AKT phosphorylation in *Smarcb1* deficient cells and to characterize *Smarcb1* dependent effects on post-transcriptional regulation, we conducted a comprehensive proteomic analysis of *Smarcb1* dependent changes in protein phosphorylation.

*Smarcb1* deficiency affected the phosphorylation of many proteins (Fig. 1). A systematic analysis of the phospho-proteomic data indicated differential activation of multiple kinases and pathways involved in regulation of cell survival and proliferation (Table 1), which are generally in agreement with our initial observation on persistent activation of AKT in *Smarcb1* deficient cells. Yet, the analysis of such data at the phosphorylation site level is confined by the limited biological information available on many of the identified the phosphorylation sites, as a result the significance of many intriguing observations remains to be explained (for example: differential phosphorylation of various nuclear pore complex proteins, centromeric proteins or lamins (see supplementary tables and figures)).

Focusing on the differential response to serum starvation between *Smarcb1* deficient and proficient cells, we identified several protein networks whose post-transcriptional regulation is altered in *Smarcb1* deficient cells (Figs. 2, 4, Additional file 2: Figure S1). The strong enrichment for cell cycle proteins among proteins that remain phosphorylated following serum withdrawal exclusively in *Smarcb1* deficient cells (Fig. 4), is in accordance with our findings on sustained proliferation of *Smarcb1* deficient cells cultured under serum starvation [14].

Regardless of serum conditions, the analysis reveals *Smarcb1* dependent phosphorylation of actin cytoskeleton and of focal adhesion proteins. Correspondingly, we find the actin skeleton of *Smarcb1* deficient cells to be diffuse and unstructured and lack stress fibers when compared to *Smarcb1* proficient cells, along with a gross difference in the number, size and distribution of focal adhesion sites (Fig. 3). Concurrently, *Smarcb1* deficient cells demonstrate an altered morphology and a reduced adhesiveness which are consistent with the changes described. *Smarcb1* expression has been previously linked to alterations in the regulation of cytoskeletal components, migration and adhesion [43, 44].

The phosphoproteomic results suggested that in *Smarcb1* deficient tumor cells phosphorylation of ErbB signaling cascade and EGFR itself persists even upon serum withdrawal (Fig. 4 and Table 1). These results were confirmed by western blot that demonstrated higher EGFR phosphorylation specifically in *Smarcb1* deficient cells (Fig. 5a). Accordingly, higher levels of total EGFR correlating to transcriptional de-repression of Egfr are observed in *Smarcb1* deficient cells (Fig. 5b). These findings suggest that EGFR activation is mediated by transcriptional upregulation of the receptor. Moreover we find *Smarcb1* mediate transcriptional inhibition of the ErbB3/HER3 receptor. This receptor is a kinase dead receptor, incompetent in promoting downstream signaling, yet heterodimers of ErbB2/HER2-ErbB3/HER3 have a potent signaling competence observed in many neoplasms [32]. As such, this de-repression of ErbB3/HER3 in *Smarcb1* deficient cells may be an additional mechanism for ErbB downstream signaling in MRT and AT/RT. Inhibition of EGFR kinase activity reduced AKT phosphorylation, indicating that it drives the activation of AKT in *Smarcb1* deficient cells. We further demonstrate the effectiveness of selective EGFR signaling inhibitors on the proliferation of *Smarcb1* deficient cells, which show increased sensitivity to Lapatinib and Gefitinib compared to *Smarcb1* proficient cells (Fig. 6). Several studies in human Rhabdoid tumor cells have demonstrated Lapatinib and Gefitinib as highly effective in inhibition of proliferation, consistent with high levels of EGFR / ErbB expression and signaling [30, 45, 46]. Taken together, our results reproduce these findings and reinforce the possibility of targeted EGFR/ErbB therapy in MRT and AT/RT patients. Moreover, we demonstrate that EGFR activation is a consequence of *Smarcb1* deficiency, suggesting that additional tumors with a mutation in *Smarcb1* or in other SWI/SNF subunits may be susceptible to EGFR/ErbB inhibitors.

Oncogenic transformation is considered to occur through a stepwise multiple-hit process, however several recent studies that examined the genome of MRT and AT/RT demonstrated exceptionally low mutation rates in both neoplasms. Indeed, when analyzing point mutations, copy number alterations or chromosomal rearrangements, all recurrent genetic aberrations were found to be limited to the *SMARCB1* locus [8–11]. Because SMARCB1 is a core component of the SWI/SNF chromatin remodeling complexes which function as transcriptional co-regulators, the low mutation rate, together with the very early onset of these tumors, raise the possibility that *SMARCB1* inactivation alone may be sufficient to drive multiple changes that promote cell transformation.

The networks we identify here and the experimental findings from our system are in line with this intriguing idea. This as they demonstrate how deficiency for *Smarcb1* results in profound transcriptional and post transcriptional deregulation, which alter the cell's response to external stimuli, its proliferative capacity and the way it interacts with the environment, in so promoting the acquisition of cancer hallmarks.

## Conclusions

The results demonstrate activation of EGFR in *Smarcb1* deficient murine rhabdoid cells lines which stems from *Smarcb1* dependent transcriptional de-repression of Egfr and possibly ErbB3/HER3. Concurrently, downstream activation of the AKT and ERK signaling cascades is evident in the tumor cells, in line with our previous findings. In accordance we find that small molecule EGFR inhibitors (specifically Gefitinib and Lapatinib) hinder the proliferation of *Smarcb1* deficient rhabdoid cells and may prove beneficial in clinical settings.

## Materials and methods

### Cell line establishment and culture

The establishment and characterization of Rhabdoid tumor cell lines 167 and 365, as well as the re-introduction of *Smarcb1* was previously described [14]. Cells were grown in DMEM supplemented with 10 % Hyclone fetal bovine serum (FBS), penicillin (50 mg/ml), streptomycin (50 mg/ml), 2 mM L-Glutamine, 0.1 nM non-essential amino acids, 0.1 mM β-Mercaptoethanol and 1 mM sodium pyruvate. For serum starvation conditions, cells were washed twice in PBS before being transferred to medium containing 0.1 % FBS. Gefitinib (Cell signaling, Cat. No. #4765), Lapatinib (Santa Cruz, Cat. No. sc-202205) and AKT inhibitor 1/2 (Calbiochem, Darmstadt, Germany, AKT inhibitor VIII No. 124018) were added in the indicated concentrations.

### Growth curves

WST-1 (Roche, Cat. No. 11–644–807–001) reagent was used with the standard protocol. Briefly, 1000 cells were plated in triplicates in a 96-well plate and cultivated for the indicated time. At each time point, 10 μl of WST-1 were added to 100 μl of growth medium and incubated for an hour. Plate was read at 480 nm with the background absorbance at 690 nm.

### Phosphoproteomic analysis

365 *Smarcb1* proficient and 365 pMIG deficient cells were SILAC labeled by culturing them for 10 population doublings in SILAC-DMEM (deprived of lysine and arginine), supplemented with 10 % dialyzed FCS and heavy, medium or light labeled lysine and arginine (lys0/arg0; lys4/arg6; lys8/arg10). Following verification of amino acid incorporation, during the experiments, cells were transferred to the same SILAC culture medium, supplemented with 10 % FCS or 0.1 % FCS over-night as illustrated in Fig. 1a. Proteins were extracted using SDS lysis buffer containing; 4 % SDS, 0.1 M DTT, 0.1 M Tris–HCl pH 7.5. Trypsin digestion was performed following the FASP protocol [47] and was followed by strong cation exchange and titanium-dioxide phosphopeptide enrichment as previously described [48].

Mass spectrometric analysis was performed on the EASY-nLC high performance liquid chromatography coupled to the LTQ-Orbitrap Velos mass spectrometer (Thermo Scientific), using data-dependent HCD fragmentation of the top 10 peptides from each MS scan. Raw MS files were analyzed with the MaxQuant software and included phospho(STY) as a variable modification. Data were filtered to have 1 % FDR on the peptide and protein levels. Data analysis was performed on the phospho(STY) sites table. Significance B calculation (based on overall distribution of the SILAC ratios and peptide intensity) was used to extract significantly changing phosphosites, with a *p*-value threshold of 0.05.

### Network analysis

Proteins found to differentially respond to serum withdrawal between v proficient and deficient cells were inputted to identify functional networks. Networks were predicted using the String database [49] with a cut-off for high confidence interactions (>0.9) based on co-occurrence, co-expression, experiments and databases. Resulting networks were visualized using the Cytoscape platform [50].

### Kinase target enrichment analysis

We utilized the data available in the kinase target database from Phosphositeplus [22] to define kinase target sets at residue level, this for residues that are defined as phosphorylated by a specific mouse kinase in mouse cells. We then applied the GSEA algorithm to search for leading edge enrichment of kinase target sets in the pre-ranked phosphoproteomic data from *Smarcb1* proficient versus deficient cells under low serum or from *Smarcb1* proficient cells grown under high serum versus serum starvation.

### Protein extraction and Western blot analysis

proteins were extracted using a Triton based buffer (0.5 % Triton, 300 mM Sucrose, 100 mM NaCl, 10 mM PIPES, 3 mM MgCl_2_*6H_2_O, 5 mM EDTA) supplemented with 1 μM DTT, 1 μM PMSF, 1 μM Pepstatin, 1 μg/ml Aprotenin, 0.5 μg/ml Leupeptin and phosphatase inhibitor cocktail 2 (Sigma, Cat. No. P5726). Following 10 min on ice the lysate was centrifuged at 14,000 rpm and the pellet discarded. Antibodies used for detection in western blot are as follows: Anti-phospho EGFR Y1092 (Abcam, 1:1000 Cat. No. ab40815), Anti-EGFR (Abcam, 1:1000 Cat. No. ab2430), anti-phospho AKT S473 (Cell signaling, 1:1000, Cat. No. 4058), Anti-AKT (Cell signaling, 1:1000, Cat. No. 11E7), anti-paxillin (Santa Cruz, 1:200, Cat. No. sc-136297), anti-beta-Actin (Abcam, 1:1000,Cat. No. ab6276), Strepavidin coupled HRP (Jackson Immunoresearch Laboratories, 1:1000). Secondary antibodies coupled to horseradish peroxidase (Jackson Immunoresearch Laboratories).

### Adhesion assay

As described in [21]. 80,000 cells from each cell line were plated in a 24 well plate in triplicates and allowed to adhere for the indicated time. The cells were then gently washed and stained with 0.5 ml 0.1 % crystal violet dissolved in 10 % acetic acid. The portion of the adhered cells was extrapolated from a standard curve prepared for each cell line concomitantly, where relative fractions from 0 % – 100 % of 80,000 cells were plated and allowed to adhere for several hours before staining.

### Immunostaining

Cells were plated on 18 mm sterile coverslips and allowed to adhere overnight. For Phalloidin cell were gently washed in PBS++ before being fixed in 3.7 % paraformaldehyde (PF) in PBS for 10 min. Following fixation cells were permeabilized in 0.5 % triton in PBS. Following several washed coverslips were stained with the Texas-Red-phalloidin (Invitrogen, 0.5U/ml, Cat. No. T7471) and DAPI (Roche, 6 μg/ml, Cat. No. 10–236–276–001) for two hours before being mounted on slides with vecta-shield. For Paxillin immunostaining, permeabilization with 0.5 % triton in 3.7 % PF in PBS with 5 % sucrose for 5 min preceded fixation for 25 min in 3.7 % PF in PBS. Following several washes and blocking with 10 % FCS in PBS for an hour, coverslips were stained with anti-paxillin (Santa Cruz, 1:200, Cat. No. sc-136297) followed by fluorescent secondary (Jackson Immunoresearch Laboratories) and DAPI before being mounted. Images were collected on a Nikon TE-2000 (Nikon, Melville, NY, USA) inverted microscope and processed using NIS-elements software (Nikon). Identical camera and microscope settings were employed to allow valid comparison between images of *Smarcb1* deficient and proficient cells.

### RNA extraction, reverse transcription and real-time PCR

All performed using standard techniques and kits as described in [51]. Primers used for expression analysis are as follows: Egfr; F': ACACTGCTGGTGTTGCTGAC R': TTGGGTGAGCCTGTTACTTG Erbb2; F': GCAGTGATCATCATGGAGCTG R': AGGTGGGTCTCAGGACTGG Erbb3; F': GTGCTGGGTTTCCTTCTCAG R': TCTGGTACTGGTTGTCAGCATC Erbb4; F': GACTTGCCAAAAATGAAGCTG R': TGCTGTTCCAGGTCAGAGAG Egf; F': CAAACGCCGAAGACTTATCC R': TTTGGCCAGTCCTCTTGTTC Errf1; F': AGCGAGCAGAGAGAAAGAGC R': ACTCTGGGATGCCTTCAAAT Beta-Actin; F': TTTTGTGTCTTGATAGTTCGCCA R': GCCGTTGTCGACGACCAG

### Errfi1 cloning

The MS2-HBTH Biotin tag was cloned by PCR from the pQCPX MS2-HBTH vector, generously provided by M. Waterman [52], using the primers: F': ACTGGCTAGCTCTCATTAATGATGGGTGG and R': ACTGGCTAGCATCCGCGGCCGCGCATG. The PCR product was restricted with NheI and ligated into the SpeI site in the pSIN-EF2-Nanog vector, which was formerly restricted with BamHI and self-ligated in-order to excise Nanog. The MS2-HBTH biotin tag was subsequently cloned from the pSin EF2-MS2-HBTH constructed backbone plasmid using the primers: F': ACTGGTCGACCATCATCACCACCATCATGAC and R': ACTGCTCGAGCTCATTAATGATGGTGGTGATG. The PCR product was restricted with SalI and XbaI and inserted into the pHAGE retroviral vector restricted with SalI.

cDNA from SMARCB1 proficent 167 cell line was used to PCR amplify Errfi1 transcript using the following primers; F': ATGCGCGGCCGCATGTCAACAGCAGGAGTTGC R': ATGCGTCGACTGGAGAAACCACGTAGGATAA. The resulting amplicon was inserted into the pHAGE-HBTH vector (described previously) between the Not1 and Sal1 restriction sites. The resulting plasmid was sequenced to ensure correct amplification and insertion. For generation of viral vectors, plasmids were co-transfected with VSVG and PHR into 293 T cells using the jetPEI® transfection reagent (Polyplus, CA, USA). Infections were carried out for 2 sequential days with 8 μg/ml Polybrene followed by selection with Blasticidin.

## Additional files

Additional file 1: Table S1 and S2. Mass-spectroscopy peptide ratios, attached as Excel files. (ZIP 5595 kb) Additional file 2: Figure S1 and S2. Functional protein networks based on differential response to serum withdrawal between *Smarcb1* proficient and deficient cells. **Figure S2.** ERRFI1 over-expression in *Smarcb1* deficient cells is insufficient in inhibition of EGFR or AKT activation. (PDF 879 kb)
