# Erratum to: ‘Phosphoproteomic analysis reveals *Smarcb1* dependent EGFR signaling in Malignant Rhabdoid tumor cells’

**DOI:** 10.1186/s12943-016-0498-2

**Published:** 2016-02-12

**Authors:** Jonatan Darr, Agnes Klochendler, Sara Isaac, Tamar Geiger, Amir Eden

**Affiliations:** 1 Department of Cell and Developmental Biology, The Alexander Silberman Institute of Life Sciences, The Hebrew University of Jerusalem, Jerusalem, Israel; 2 Department of Human Molecular Genetics and Biochemistry, Sackler Faculty of Medicine, Tel Aviv University, Tel Aviv, Israel

Unfortunately, the original version of this article [1] contained an error. An author’s name was spelt incorrectly. It was spelt as Tami Geiger when it should have been spelt as Tamar Geiger instead which is the correct spelling and included here in this erratum.

## References

[1] Jonatan D, Agnes K, Sara I, Tamar G, Amir E (2015). Phosphoproteomic analysis reveals *Smarcb1* dependent EGFR signaling in Malignant Rhabdoid tumor cells. Mol Cancer.

