# Supplementary material for: Phosphoproteomic analysis reveals Smarcb1 dependent EGFR signaling in Malignant Rhabdoid tumor cells
Source: Mol Cancer. 2015 Sep 15;14:167. doi: 10.1186/s12943-015-0439-5 (PMC4570560; doi:10.1186/s12943-015-0439-5)
Supplement: Additional file 2: Figure S1 and S2. — Functional protein networks based on differential response to serum withdrawal between Smarcb1 proficient and deficient cells. Figure S2. ERRFI1 over-expression in Smarcb1 deficient cells is insufficient in inhibition of EGFR or AKT activation. (PDF 879 kb) [file 12943_2015_439_MOESM2_ESM.pdf]

Phosphoproteomic analysis reveals *Smarcb1* dependent EGFR signaling in Malignant Rhabdoid tumor cell lines

Jonatan Darr, Agnes klochendler, Sara Isaac, Tami Geiger and Amir Eden*

SUPPLEMENTARY INFORMATION

Supplementary Tables 1 and 2: Mass-spectroscopy peptide ratios, attached as Excel files.

Supplementary Figure 1: Functional protein networks based on differential response to serum withdrawal between *Smarcb1* proficient and deficient cells.

Supplementary Figure 2: ERRFI1 over-expression in *Smarcb1* deficient cells is insufficient in inhibition of EGFR or AKT activation.

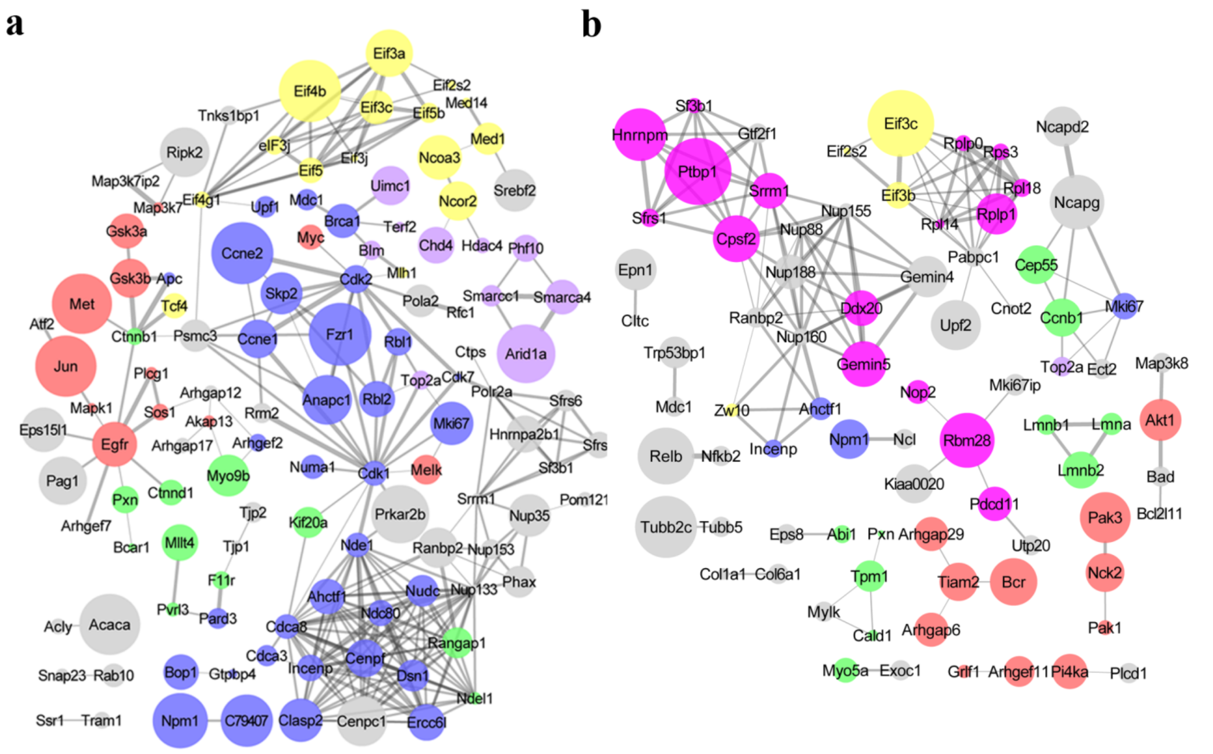

Supplementary Figures 1: Functional protein networks based on differential response to serum withdrawal between *Smarcb1* proficient and deficient cells. A: Proteins which remain phosphorylated in *Smarcb1* deficient cells upon serum withdrawal but lose their phosphorylation in *Smarcb1* proficient cells (blue group from figure 4). **B:** Proteins which lose phosphorylation specifically in *Smarcb1* deficient cells upon serum withdrawal (gray group from figure 4). Networks were constructed using the String database (52) with a cut-off for high confidence interactions based on co-occurrence, co-expression, experiments and databases. Resulting networks were visualized using the Cytoscape platform (53). Only highest confidence score (>0.9) interactions as calculated by String V9.1 are portrayed. Size of node is correlated with the degree of differential regulation between *Smarcb1* proficient and deficient cells. Blue – Cell cycle; Red – Kinase, ErbB, Rho GTPase; Green – Cytoskeleton; Violet – RNA processing; Light violet – chromatin modification; Yellow – initiation factor

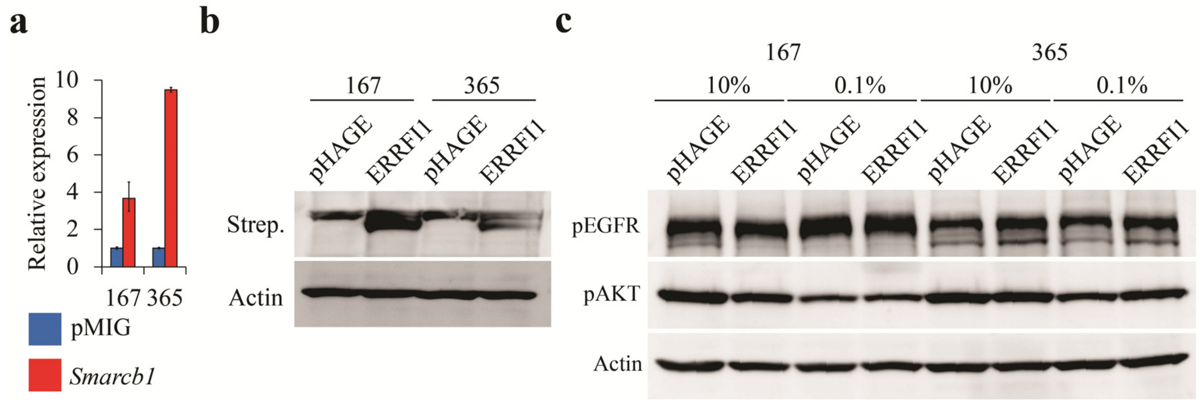

Supplementary Figure 2. ERRFI1 over-expression in *Smarcb1* deficient cells is insufficient in inhibition of EGFR or AKT activation. (a) Quantitative-rtPCR demonstrating induction of ERRFI1 upon *Smarcb1* re-expression. **(b)** Over-expression of biotin-tagged ERRFI1 in *Smarcb1* deficient cells. **(c)** EGFR and AKT phosphorylation unaltered following ERRFI1 over-expression.
